# Ultrasensitive response motifs: basic amplifiers in molecular signalling networks

**DOI:** 10.1098/rsob.130031

**Published:** 2013-04

**Authors:** Qiang Zhang, Sudin Bhattacharya, Melvin E. Andersen

**Affiliations:** Center for Dose Response Modeling, Institute for Chemical Safety Sciences, The Hamner Institutes for Health Sciences, Research Triangle Park, NC 27709, USA

**Keywords:** ultrasensitivity, sigmoid, response coefficient, Hill coefficient, motif

## Abstract

Multi-component signal transduction pathways and gene regulatory circuits underpin integrated cellular responses to perturbations. A recurring set of network motifs serve as the basic building blocks of these molecular signalling networks. This review focuses on ultrasensitive response motifs (URMs) that amplify small percentage changes in the input signal into larger percentage changes in the output response. URMs generally possess a sigmoid input–output relationship that is steeper than the Michaelis–Menten type of response and is often approximated by the Hill function. Six types of URMs can be commonly found in intracellular molecular networks and each has a distinct kinetic mechanism for signal amplification. These URMs are: (i) positive cooperative binding, (ii) homo-multimerization, (iii) multistep signalling, (iv) molecular titration, (v) zero-order covalent modification cycle and (vi) positive feedback. Multiple URMs can be combined to generate highly switch-like responses. Serving as basic signal amplifiers, these URMs are essential for molecular circuits to produce complex nonlinear dynamics, including multistability, robust adaptation and oscillation. These dynamic properties are in turn responsible for higher-level cellular behaviours, such as cell fate determination, homeostasis and biological rhythm.

## 2. Introduction

Cells constantly sense changes in their surrounding environment and elicit appropriate responses. These responses require information about the surroundings to be conveyed into and then processed by intracellular biochemical networks. Although cellular responses can sometimes be proportional to the environmental cues, biological signals often propagate in a nonlinear fashion, resulting in altered amplitude, duration and phase [1–5]. Ultrasensitivity is a form of nonlinear signal processing where a small fractional change in the input signal is amplified, producing a larger fractional change in the output response [6–8]. As a result, the output is not a proportional function of the input, and viewed on a double-linear scale the input–output (I/O) curve of an ultrasensitive response generally has a sigmoid appearance [9].

The term ‘amplification’ can cause confusion in biology, at times referring to qualitatively different concepts. In some cases, the term is used to refer to absolute concentration amplification, where a chemical species operating in a low molar concentration range controls another species existing in a high molar concentration range. This form of amplification is necessary for control of actuator molecules in the cellular machinery, where high abundance is needed to carry out functions on a scale significant to the cell or tissue. A primary example is the coagulation enzyme cascade that may start with a few molecules of factor XII and culminate in the activation of millions of times more fibrin molecules. In most other contexts (and also here in this review), the term ‘amplification’ means relative concentration amplification or sensitivity amplification. Weber's law, which states that sensation of the environment by an organism works by recognition of the relative change in the perceived signal, also appears to operate at the molecular signalling level [10]. The magnitude of relative (or fold) change in protein signalling in response to extracellular stimuli can be more robustly retained than the absolute change among a population of isogenic but otherwise heterogeneous cells [11], or in the presence of perturbations that cause absolute protein level changes [12]. More importantly, phenotypic outcomes such as embryonic development respond more consistently to signals that retain the same fold, rather than absolute, change [12], suggesting that cells choose to interpret relative changes in the level of signalling molecules as the *bona fide* instructing signals. The role of ultrasensitivity is to amplify these relative changes at appropriate locations in molecular signalling networks.

Signal amplification through basic circuit units—referred to here as ‘ultrasensitive response motifs’ (URMs)—is essential for enabling multiple cellular dynamics. In the absence of URMs, a signalling cascade is not even likely to output a linear response owing to saturation of binding. Amplification via URMs can make up for the amplitude loss and help maintain the dynamical range of the original signal. A highly ultrasensitive motif can function as a switch, transforming a continuous signal into an all-or-none response. The functional importance of signal amplification, as engendered by URMs, can be best understood by studying complex nonlinear dynamics, such as bistability, adaptation and oscillation. These dynamics are fundamental to a multitude of integrated cellular functions, including proliferation, differentiation, homeostasis and biological rhythm [13–15]. URMs confer the nonlinearity necessary for these dynamical properties to be rendered by properly structured molecular networks. In this sense, URMs are the biochemical equivalents of current- or voltage-amplifying transistors, the fundamental building component of modern analogue and digital electronic devices [16].

We begin the review by first introducing response coefficient as the measure of ultrasensitivity. We discuss how it is related to the Hill function that is often invoked to approximate sigmoid responses. We then extensively cover six distinct types of URMs. For each URM, we provide an intuitive explanation of the signal-amplifying mechanism as well as a simple mathematical model to quantitatively illustrate the chemical kinetics underlying amplification. Numerous biological examples are covered to demonstrate the ubiquity of ultrasensitivity in molecular signalling networks. In §5, we illustrate, with feedback circuits capable of bistability, adaptation and oscillation, the critical role of ultrasensitivity in enabling complex dynamical behaviours. Mathematical models discussed in the review are available in SBML format as electronic supplementary material.

## 3. Ultrasensitivity

### 3.1. Response coefficient, ultrasensitivity and sigmoid curve

The sensitivity of the steady-state stimulus–response function of a target molecular species *Y* that is directly or indirectly regulated by a signalling molecular species *X* can be quantified by the ratio of the fractional changes in *Y* and *X*:3.1
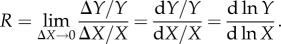
*R* is known as response coefficient in metabolic control analysis [17,18] and as logarithmic gain (‘gain’ for short) in biochemical systems theory [19,20]. When *R* = 1, the response is proportionally linear. When *R* > 1, a small percentage increase/decrease in *X* results in a larger percentage increase/decrease in *Y*, indicating a response more sensitive than the linear case. Ultrasensitivity is thus defined as a response that has a response coefficient significantly greater than 1. Conversely, when 0 < *R* < 1, a small percentage increase/decrease in *X* results in an even smaller percentage increase/decrease in *Y*, which is referred to as a subsensitive response. When *X* inhibits *Y*, *R* has a negative value, and the conditions |*R*| > 1 and 0 < |*R*| < 1 define ranges of ultrasensitivity and subsensitivity, respectively.

**Figure 1.**
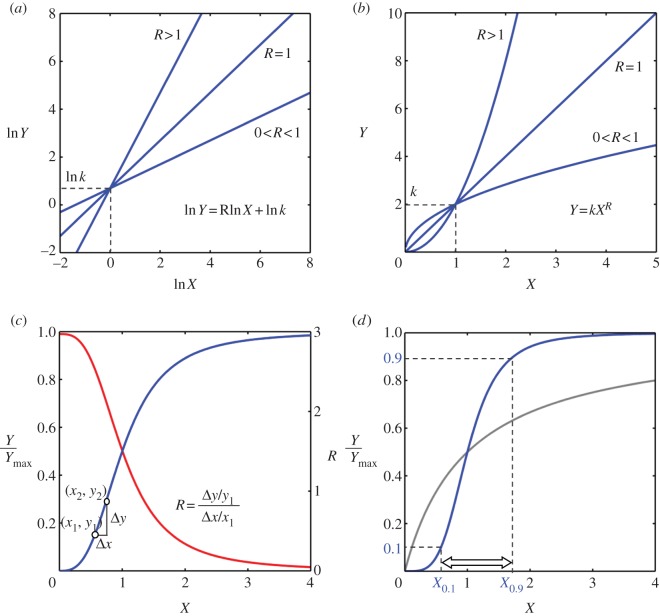
Response coefficient, shape of ultrasensitive response curve and Hill function. (*a*) On a log–log scale, if response coefficient *R* remains constant, proportional, ultrasensitive or subsensitive responses are straight lines of slope of 1, greater than 1 or less than 1, respectively. (*b*) On a linear scale, if response coefficient *R* remains constant, a proportional response (*R* = 1) is a straight line; an ultrasensitive response (*R* > 1) appears as a curve concave upward and a subsensitive response (0 < *R* < 1) appears as a curve concave downward. (*c*) A typical saturable ultrasensitive stimulus–response has a sigmoid appearance (blue curve, left *y-*axis). Not all regions of the sigmoid curve are ultrasensitive (i.e. capable of percentage amplification). The actual ultrasensitive region corresponds to the range of *X* where the local response coefficient *R* (red curve, right *y*-axis) is greater than 1. (*d*) Hill function (blue curve) is frequently used to represent an ultrasensitive response. The global steepness of the Hill curve is defined by the Hill coefficient *n* (see equation 3.5), which quantifies the relative fold change in the level of *X* that produces from 10 to 90 per cent of the maximum response. The Michaelis–Menten response is plotted as a reference (grey curve).

If *R* remains constant as *X* varies, the steady-state relationship between *Y* and *X* is described by the equation3.2
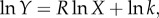
where *k* is a constant. Transformed to a linear scale, it becomes3.3
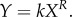
For *R* > 1 (i.e. an ultrasensitive response), the *Y* versus *X* stimulus–response curve is concave upward; for 0 < *R* < 1 (i.e. a subsensitive response), the curve is concave downward (figure 1*a*,*b*).

For an ultrasensitive response, as long as *R* remains constant as *X* varies, the shape of the stimulus–response curve would remain upward concave. Although ultrasensitivity is a form of nonlinear amplification, as far as relative (percentage) change is concerned, the amplification can be regarded as ‘linear’ as long as *R* remains constant, as shown on the log–log scale (figure 1*a*). However, the response coefficient of a signalling cascade rarely stays constant with respect to the input signal. An important feature of biochemical signalling is saturation (i.e. when the input signal is sufficiently strong, the response tends to level off). Thus, for an ultrasensitive motif that is saturable, the response coefficient would decrease from *R* > 1 to *R* < 1 and to *R* ≈ 0 as the input signal intensifies. Correspondingly, the upward concave curve would gradually grow less steep as it moves first into a downward concave phase and finally into a plateau, forming a sigmoid curve overall (figure 1*c*). Therefore, ultrasensitivity is typically characterized by a full-range steady-state response that is sigmoidally shaped on a linear scale and relatively steeper than the rectangular hyperbola characterizing the Michaelis–Menten type of response [9,21].

### 3.2. Hill function

An ultrasensitive response is often empirically approximated by the Hill function, which was initially derived from the study of oxygen binding to haemoglobin [22]:3.4
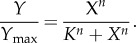
*Y*_max_ is the maximum activity of *Y*, *K* is the level of *X* producing a response of 50 per cent of *Y*_max_ and *n* is the Hill coefficient determining the steepness of the curve. A measure of the Hill coefficient *n* is provided by3.5
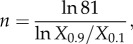
where *X*_0.1_ and *X*_0.9_ are the levels of *X* associated with, respectively, 10 per cent and 90 per cent of the maximum response (figure 1*d*). The Hill coefficient *n* thus numerically quantifies the steepness of a sigmoid curve relative to the hyperbolic Michaelis–Menten curve, where *X*_0.9_/*X*_0.1_ = 81 and *n* = 1. A higher *n-*value means a shorter distance between *X*_0.1_ and *X*_0.9_, and hence a steeper sigmoid curve. Unlike the response coefficient, which defines the local ultrasensitivity (steepness) of the stimulus–response curve, the Hill coefficient provides a global measurement of the overall steepness of the curve. The response coefficient of the Hill function is given by Goldbeter & Koshland [8] and Sauro [23]:3.6
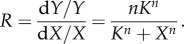
When *X* is very small compared with *K*, *R* approximately equals *n*. Thus, the Hill coefficient, a metric describing the global steepness of the Hill function, is equivalent to the response coefficient at low input levels.

**Figure 2.**
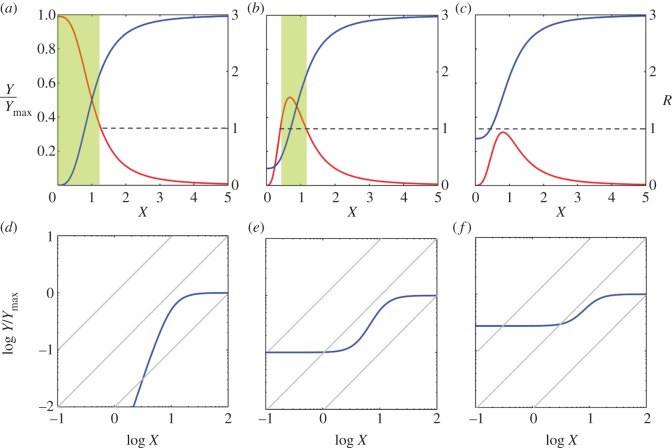
Effect of basal activity of output on ultrasensitivity. (*a*–*c*) Solid blue curves describe the *Y*/*Y*_max_ (left *y*-axis) versus *X* stimulus–response as represented by equation 3.7, and red curves are the corresponding response coefficient *R* (right *y*-axis). As the basal activity of *Y* (*Y*_0_) increases (from (*a*) through (*c*)), the maximum response coefficient decreases. The actual ultrasensitive regions are marked by the shaded areas, which have response coefficients of more than 1. The sigmoid response curve in (*c*) loses ultrasensitivity completely. (*d*–*f*) Blue stimulus–response curves in (*a*–*c*) re-plotted on a log–log scale, respectively. The degree of ultrasensitivity can be visually assessed by comparing the slopes of the stimulus–response curve with a series of straight lines of slope of unity (grey lines). Ultrasensitivity is indicated when a section of the curve is steeper than the straight lines.

In many signalling cascades, the output may already have some basal activities even in the absence of the input signal. This situation can be described by the equation3.7
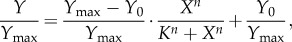
where *Y*_0_ is the basal activity of *Y*. The presence of the basal activity desensitizes the response, particularly for low input levels [7]. After all, the sensitivity of a response, as measured by the response coefficient, is related to the percentage rather than absolute change. When *Y*_0_ is sufficiently large, ultrasensitivity can disappear completely even though the response curve still remains fairly sigmoid (figure 2*a*–*c*). Thus, response coefficient is always a more reliable measure for ultrasensitivity than Hill coefficient, especially for stimulus–response curves that cannot be easily fitted with Hill functions. A simple way to visually gauge the degree of ultrasensitivity is to compare the slopes of the response curve with straight lines of slope of unity in a log–log plot (figure 2*d*–*f*).

## 4. Ultrasensitive response motifs

The empirical description of ultrasensitive response by the Hill function does not provide necessary mechanistic insight, and is sometimes inadequate to delineate the exact shape of an actual stimulus–response curve. Ultrasensitivity must arise from the kinetics of specific biochemical interactions. Based on knowledge of known interactions, both theoretical and experimental studies in the past several decades have uncovered a number of URMs, which can be grouped by and large into six common categories: (i) positive cooperative binding, (ii) homo-multimerization, (iii) multistep signalling, (iv) molecular titration, (v) covalent modification cycle (zero-order ultrasensitivity) and (vi) positive feedback. Although the topic of ultrasensitivity was previously reviewed by others and us [8,9,24,25], here we attempt to provide a much more comprehensive and up-to-date coverage of these motifs, by elucidating their specific ultrasensitive mechanisms and including relevant biological examples.

### 4.1. Positive cooperative binding

**Figure 3.**
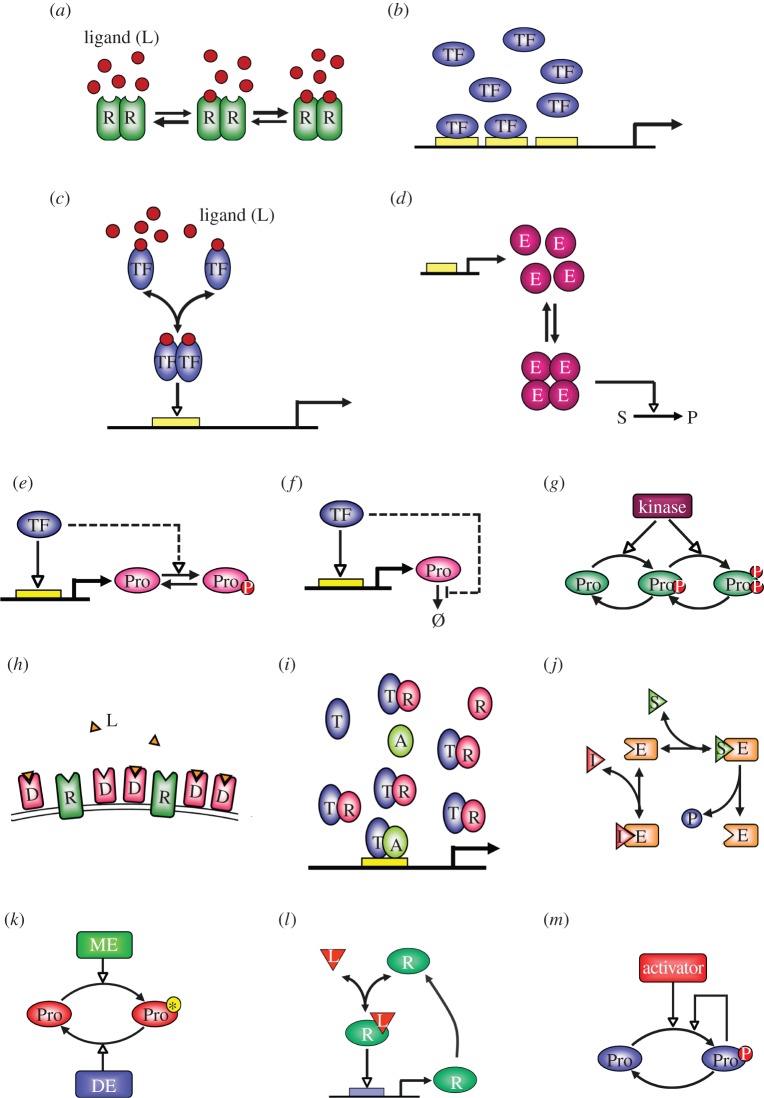
Illustrations of ultrasensitive response motifs. (*a*) Positive cooperative binding between ligand L and multimeric (two subunits illustrated) receptor R. The sequential increase in binding affinity is indicated by changes in the thickness of transition arrows. The overall activity of R is proportional to its percentage occupancy by L. (*b*) Positive cooperative binding between TF and multiple response elements in gene promoters. The transcriptional activity of the promoter is proportional to its percentage occupancy by TF. (*c*) Homo-multimerization of TFs to transcriptionally active multimers. Illustrated are TFs activated by ligand binding to form homo-dimers, which gain affinity for DNA promoter. (*d*) Many inducible enzymes catalysing xenobiotic detoxification or metabolic reactions function as homo-multimers. Here, inducible enzyme monomers E associate with one another to form homo-tetramers, which are fully enzymatically active to convert substrate S to product P. (*e*) Synergistic multistep signalling where a TF directly increases the abundance of the target protein (Pro) through transcriptional induction, and indirectly increases the activity of Pro (dashed line) through processes such as induction of a kinase (not shown) that phosphorylates and thus activates Pro. (*f*) A TF may increase the abundance of the target protein Pro through direct transcriptional induction, and indirectly by inhibiting degradation of Pro (dashed line) by inducing factors (not shown) that stabilize Pro. (*g*) Multisite phosphorylation of protein substrate Pro by the same kinase in a non-processive manner is a common multistep signalling ultrasensitive motif. (*h*) Molecular titration with decoy or dominant-negative receptor D competing with wild-type receptor R for ligand L. (*i*) Molecular titration with transcriptional repressor R competing with activator protein A for transcription factor T. (*j*) Molecule I competitively inhibits enzyme E, preventing it from binding to substrate S and catalysing the reaction. (*k*) Zero-order ultrasensitivity by covalent modification cycle. Protein substrate Pro can be reversibly modified and de-modified by modifier enzyme (ME) and de-modifier enzyme (DE). (*l*) Positive gene auto-regulation where ligand L activates receptor R, which transcriptionally upregulates its own abundance, thus forming a positive feedback loop. (*m*) Auto-catalysis where an activator, such as a kinase, phosphorylates a substrate protein (Pro). Then phosphorylated Pro can also function as a kinase to catalyse its own phosphorylation. Solid arrow head, chemical conversion or flux; empty arrow head, positive regulation; blunted arrow head, negative regulation.

Many receptor proteins exist as multimeric complexes, comprising multiple identical or similarly structured subunits. Each subunit contains one binding site for one molecule of the cognate ligand. According to the common Adair/Koshland–Nemethy–Filmer model developed for oxygen binding of haemoglobin [26–28], positive cooperative binding occurs when the receptor subunits already occupied by ligand molecules through early binding events can facilitate subsequent binding of the remaining unoccupied subunits by the ligand (figure 3*a*). This sequential increase in binding affinity can result from allosteric interactions among the subunits of the receptor. In the extreme case where the affinities of the late binding events are enormously greater than those of the early binding events, the receptor tends to reside in one of two states: either free of any ligand molecules, or fully occupied by ligand molecules. This is because once one subunit is occupied first, binding to the remaining subunits will follow suit quickly owing to the enhanced affinity. Such binding kinetics tend to give rise to a sigmoid response in terms of percentage receptor occupancy, with the degree of ultrasensitivity (or cooperativity) dependent on the total number of binding sites per receptor molecule and the extent of increment in binding affinity for sequential binding events. In the electronic supplementary material, a mathematical model of ligand–receptor interaction is provided to illustrate the ultrasensitive mechanism of positive cooperative binding (motif 1).

Positive cooperative binding can provide ultrasensitive signalling for second messengers. Activation of protein kinase A (PKA) requires binding of cyclic adenosine monophosphate (cAMP) to its regulatory subunit, which contains two cAMP-binding sites. It was demonstrated that cAMP binding to the regulatory subunit proceeds with positive cooperativity, giving rise to a sigmoid PKA activation curve with a Hill coefficient of 1.4–1.6 [29,30]. Ca^2+^ as a second messenger is also capable of positive cooperative binding. Signalling information carried by Ca^2+^ is routinely relayed through Ca^2+^-binding proteins such as calmodulin, cytosolic phospholipase A_2_ and calretinin, which contain multiple Ca^2+^-binding sites. *In vitro* evidence indicates that occupancy of these sites by Ca^2+^ often exhibits positive cooperativity of various degrees [31–34]. Ultrasensitivity arising from positive cooperative binding in the second messenger system may serve as an amplifying mechanism to ensure unattenuated signal transduction.

Cooperative binding can also occur between transcription factors (TFs) and *cis*-regulatory response elements in gene promoters (figure 3*b*). In the invertebrate and vertebrate genomes, multiple response elements for a particular TF are frequently clustered together [35–37], making cooperative binding possible through allosteric interactions between adjacent elements. The cooperativity can also be facilitated by protein–protein interaction between free and DNA-bound TF molecules [38,39]. Bicoid, a morphogenic TF, forms a concentration gradient along the anterior–posterior (A–P) axis in the early *Drosophila* embryo [40]. Bicoid can bind to multiple copies of a *cis*-acting consensus DNA sequence in a highly cooperative manner, contributing to a sharp, nearly step-like expression distribution along the A–P axis of some target genes, such as hunchback, which direct embryonic pattern formation [41–43]. Heat shock factors (HSFs), activated by rise in temperature to induce heat shock and chaperon proteins, also appear to interact with its target gene promoters in a highly cooperative manner [38,44,45].

### 4.2. Homo-multimerization

Many proteins function in the form of homo-multimers. In the process of protein homo-multimerization (or homo-oligomerization), identical monomers reversibly associate with one another to form higher-order multimers that usually possess full functional activity. According to mass action kinetics, the formation rate of the multimer varies as a power function of the monomer concentration, with the exponent equal to the order of multimerization (see motif 2 in the electronic supplementary material). As a result, a linear increase in the concentration of the free monomer would drive an ultrasensitive increase in the steady-state concentration of the multimer. In theory, the response coefficient can be as high as 2 for homo-dimerization, 3 for homo-trimerization, and so on.

Protein homo-multimerization is a common step in signal transduction, gene regulatory and metabolic networks. Examples are formation of dimeric, trimeric or tetrameric receptors, TFs and holoenzymes. Activation of cell membrane receptors belonging to the receptor tyrosine kinase (RTK) family requires receptor homo-dimerization after ligand binding. A kinetic study by Park *et al*. [46] on one of the RTKs, the platelet-derived growth factor (PDGF) receptor, demonstrated that phosphorylation of the receptor, when stimulated by PDGF ligands, exhibited a sigmoid response with Hill coefficient of 1.55. Using mathematical modelling, they suggested dimerization between two monomeric ligand–receptor complexes as a possible mechanism behind the observed sigmoid response. By contrast, the epidermal growth factor (EGF) receptor showed negative cooperativity with its cognate ligand, a phenomenon resulting from sequential ligand-binding kinetics in which the affinity of the second EGF ligand-binding event (to singly liganded receptor dimers) is allosterically weakened [47].

As an essential step towards their genomic action, steroid hormone receptors associate into homo-dimers upon ligand binding (figure 3*c*) to gain high affinity for the hormone response elements in target genes [48–50]. *In vitro* binding assays with oestradiol, progesterone and their cognate receptors demonstrated that the monomer–homodimer kinetics can lead to ultrasensitive responses in the formation of ligand-bound receptors [51,52]. Many TFs activated by mechanisms other than ligand binding also function as high-order homo-multimers. Although remaining to be validated experimentally, activation of multimeric TFs is expected to exhibit ultrasensitivity if the activating signal ultimately drives more monomers to associate into multimers as opposed to just modifying constitutively expressed, pre-existing multimers. Examples of homodimeric TFs are tonicity-responsive enhancer-binding protein (TonEBP or NFAT5) mediating osmotic stress response [53], members of the signal transduction and activator of transcription family [54], immediate early gene products such as c-Jun [55] and the myogenic determination factor involved in muscle lineage development [56]. Active HSF is an example of a homo-trimer [57]; OxyR, activated by oxidative stress in bacteria [58], and p53, activated by DNA damage, are homo-tetramers [59].

Many metabolic enzymes induced in cellular stress response act as homo-multimers (figure 3*d*). For instance, a suite of antioxidant proteins induced by oxidative stress are homo-dimers or homo-tetramers [60]. In particular, glutathione peroxidase and catalase, the two enzymes catalysing reactions to detoxify hydrogen peroxide, function as homo-tetramers [61,62]. Anti-stress proteins that function as homo-dimers also include metallothioneins induced by heavy metal stress to chelate metal molecules [63], and the growth arrest and DNA damage protein (GADD45) induced by genotoxic stress to repair damaged DNA and control cell growth [64,65]. Transcriptional induction of monomeric proteins and their subsequent multimerization into active enzymatic complexes play a crucial ultrasensitive role in feedback networks that cope with cellular stresses to maintain robust homeostasis [14,60]. In *Escherichia coli*, activation of glutamine synthetase (GS) by glutamine is a bicyclic cascade process involving an intermediate protein PII; it was found that PII functioning as a homo-trimer is a necessary step towards rendering the activation of GS by glutamine ultrasensitive, with a Hill coefficient as high as 6.5 *in vitro* [66].

### 4.3. Multistep signalling

Multistep signalling describes a signalling scheme where a common input signal simultaneously regulates two or more biochemical processes that synergistically activate an output response. For instance, (i) a regulatory protein may increase both the abundance and activity of a target protein, respectively, through transcriptional induction and post-translational modification; (ii) a TF may simultaneously induce the transcription of a target gene and also indirectly inhibit the degradation of its protein product; and (iii) a kinase may activate a target protein through non-processive multisite phosphorylation (figure 3*e*–*g*). In each of these signalling schemes, synergy between parallel processes is manifested ultimately as multiplicative terms in the mathematical description of the output response. As a result, ultrasensitivity would arise even if the input signal regulates each individual process linearly. In the electronic supplementary material, a mathematical model of dual regulation is provided to illustrate the multistep signalling effect (motif 3).

Non-processive (distributive) multisite protein phosphorylation by a single kinase is a common multistep signalling motif [67,68]. In this situation, only fully phosphorylated or fully dephosphorylated proteins are assumed to have the maximal activity. Ultrasensitivity arising from multisite phosphorylation can be understood by using mitogen-activated protein kinase (MAPK) as an example. Dual phosphorylation of MAPK is achieved through two separate reactive collisions (rather than a single collision) between the MAPK kinase (MAPKK, as enzyme) and MAPK (as substrate) molecules [69,70], during which single-phosphorylated MAPK is released as an intermediate product and then re-associates with MAPKK as a substrate. An increase in MAPKK concentration thus leads to (i) an increasing amount of single-phosphorylated MAPK as the substrate for the second phosphorylation reaction, and (ii) an increasing amount of kinase to catalyse the second phosphorylation reaction. As a result, the production rate of dual-phosphorylated MAPK can vary ideally as a square of MAPKK concentration, contributing to MAPK ultrasensitivity [71]. Were multisite phosphorylation achieved processively in a single collision, no ultrasensitivity would arise. MAPK ultrasensitivity is also contributed to by multisite dephosphorylation, and in this regard, it has been shown that MAPK phosphatase-3 dephosphorylates ERK2 in a non-processive manner [72].

In budding yeast, stoichiometric inhibitor of cyclin-dependent kinase 1 (Cdk1; Sic1), a protein inhibiting G1/S phase transition in the cell cycle, has to be phosphorylated on at least six sites by the Cdk in order to be ubiquitinated for degradation [73]. The multisite phosphorylation process is believed to occur in a distributive fashion (although this was recently challenged [74]) and generate a potentially ultrasensitive response that contributes to the bistable switch underlying G1 to S phase transition [75,76]. Activation of transcription factor NFAT1 requires dephosphorylation of 13 serine residues by calmodulin-dependent phosphatase calcineurin; removal of the multiple phosphate groups masks the nuclear export sequence and exposes the nuclear import sequence, allowing NFAT1 to translocate into the nucleus and become transcriptionally active [77]. Mathematical modelling predicted that if some of the dephosphorylation steps proceed distributively (i.e. with multiple association/dissociation events between calcineurin and the intermediate substrates), an ultrasensitive response with high Hill coefficient would arise [78]. This may partially explain the nonlinear induction of NFAT1 target genes observed experimentally [79]. Recently, Trunnell *et al.* [80] demonstrated that activation of Cdc25C by Cdk1, two key components involved in a bistable switch circuit responsible for entry into mitosis, exhibits a highly ultrasensitive response in *Xenopus* oocyte extracts. The ultrasensitive mechanism is attributed to multisite phosphorylation of Cdc25C by Cdk1.

While increasing the number of phosphorylation sites generally enhances the degree of ultrasensitivity, theoretical work has predicted that non-processive multisite phosphorylation alone tends to create a response with a threshold followed by a more graded change, rather than an abrupt switch [68]. To generate a switch-like response, additional mechanisms are needed, including cooperativity associated with sequential phosphorylation, competition for kinase between intermediate substrates in variously phosphorylated states, substrate sequestration, sequential rather than random phosphate processing and local kinase saturation owing to anchorage of substrates to cell membranes [67,68,78,81–83]. Another situation that may complicate signalling through multisite phosphorylation is the existence of scaffold proteins, such as those required for the MAPK cascade. Computational studies have shown that scaffold proteins can modulate MAPK activation in terms of magnitude, timing and degree of ultrasensitivity [84,85]. By physically bringing the kinases and their next-level protein substrates into close proximity, scaffold proteins can increase signalling strength and specificity. However, their existence may also diminish MAPK signalling by (i) the ‘prozone effect’, wherein excessive scaffold molecules may hold the kinases and substrates in separate non-functional complexes, and (ii) the tendency of on-scaffold kinases to be phosphorylated processively rather than distributively.

Multistep signalling is also involved in the regulation of protein activity by small signalling molecules. For example, an increase in the AMP/ATP ratio as a result of energy depletion activates AMP-activated protein kinase (AMPK) through four distinct mechanisms simultaneously. These multistep regulations include: (i) AMP allosterically activates AMPK kinase (AMPKK), which phosphorylates (i.e. activates) AMPK [86]; (ii) by binding to unphosphorylated AMPK, AMP enhances the rate of phosphorylation of AMPK by AMPKK [87]; by binding to phosphorylated AMPK; (iii) AMP reduces the rate of dephosphorylation of AMPK by phosphatases [88]; and (iv) AMP allosterically enhances the activity of AMPK as a kinase [89]. Together with some degree of zero-order ultrasensitivity, these multiple signalling steps contribute to a sigmoid activation of AMPK [90]. In a similar manner, Ca^2+^/calmodulin-dependent kinase I is also activated by Ca^2+^/calmodulin through multistep regulations [86].

Many TFs involved in cellular stress response are activated by stress signals in multiple ways. Under hypoxia, as O_2_ level decreases, proline hydroxylation of hypoxia inducible factor-1α (HIF-1α) is diminished, which stabilizes HIF-1α, leading to its accumulation [91,92]. Second, lower O_2_ level also decreases the hydroxylation of an asparagine residue of HIF-1α, leading to enhanced transcriptional activity of HIF-1α [93,94]. Together with a potential molecular titration mechanism [95], this dual regulation by O_2_ partial pressure may lead to an ultrasensitive activation of HIF-1α under hypoxia, which in turn contributes to an exponential or switch-like induction of anti-hypoxic genes such as erythropoietin [96–98]. Another example of stress activation of TF via multistep signalling is nuclear factor E2-related factor 2 (Nrf2). The cellular redox state regulates Nrf2 in at least three ways. (i) An oxidative environment in the cell tends to stabilize Nrf2 protein by inhibiting its redox-sensitive negative regulator Kelch-like ECH-associated protein 1, which is an adaptor protein for E3 ubiquitin ligase targeting Nrf2 for proteasomal degradation [99,100]. (ii) The 5′ untranslated region of Nrf2 mRNA contains an internal ribosomal entry site, which can enhance the translation of Nrf2 protein in a redox-sensitive manner [101]. (iii) Nrf2 protein itself also contains a redox-sensitive nuclear export signal that is inhibited by an oxidative intracellular environment [102]. Thus, under oxidative stress, Nrf2 could accumulate in the nucleus under three synergistic forces: (i) increased protein stabilization, (ii) enhanced translation and (iii) increased nuclear retention.

### 4.4. Molecular titration

Many stoichiometric inhibitors exist in cells to scavenge signalling molecules into inactive complexes, titrating them away from downstream target molecules. Generic examples of titration (also termed as protein sequestration when titration occurs between protein molecules) are: (i) wild-type and decoy/dominant-negative receptors competing for a common ligand [103–106]; (ii) TFs dimerizing with either partner proteins to form a transcriptionally active complex or with repressor proteins to form a transcriptionally inactive complex [107–110]; and (iii) competitive enzyme inhibition (figure 3*h*–*j*). Hidden in this seemingly trivial inhibition scheme is an ultrasensitive response, which occurs when the inhibitor (the total amount of which is *I*) exists in a large quantity and the signalling molecule (the total amount of which is *S*) has a higher binding affinity for the inhibitor than for the target molecule [24,111,112]. Ultrasensitivity arises near the point where nearly all of the inhibitor molecules are ‘used up’ by forming inactive complexes with the signalling molecules. At that point, any additional small increase (*ΔS*) in the amount of the signalling molecules in the system will be almost entirely available for binding to its target molecules, thus producing a sharp increase in the formation of the active complex. Mathematically, it is straightforward to note that once the inhibitor is saturated, the fractional increase in the available signalling molecule, which is roughly equal to *ΔS*/(*S* − *I*), is always greater than the fractional increase in the total signal molecule, which is *ΔS*/*S*. Thus the ratio of the two fractions (i.e. the response coefficient) will be greater than unity, and ultrasensitivity is indicated. It is also obvious that a larger *I* denotes a larger *ΔS*/(*S* − *I*) and hence a higher response coefficient. In the electronic supplementary material, motif 4 illustrates this ultrasensitive mechanism.

A number of synthetic biology studies have provided convincing experimental evidence for ultrasensitivity generated via molecular titration. By introducing transcription factor CCAAT/enhancer-binding protein α (CEBPα) and an engineered high-affinity dominant-negative inhibitor into yeast cells, Buchler & Cross [111] demonstrated a nearly switch-like gene expression response that is consistent with ultrasensitivity predicted by molecular titration. Many long stretches of non-coding tandem repeats in the genome have long been suspected to act as repressive decoy TF-binding sites that can sequestrate free TFs [113]. Recently, by introducing plasmid arrays containing a couple of hundred of non-functional tet operators into budding yeasts, Lee & Maheshri [114] demonstrated that gene expression driven by tet-transcriptional activators can be converted into a sharp sigmoid response in the presence of these repressive binding sites. Likewise, *in vitro* occupancy of target DNA sequence by TATA-binding protein, as detected by optical DNA sensors, also exhibited switch-like responses in the presence of competing sequences [115].

Many signalling enzymes can act on two or more substrates, and sometimes competitive inhibitors also exist. In *Xenopus* oocyte extract, phosphorylation of Wee1 by Cdk1, required for interphase to mitosis transition, is ultrasensitive, a response partially arising from intermolecular competition for Cdk1 between Wee1 and some unidentified substrates [116]. Ultrasensitivity arising from intramolecular titration was recently observed in the spindle orientation signalling pathway in *Drosophila* neuroblasts, which contains (i) heterotrimeric G-protein α-subunit Gαi, (ii) Partner of nscuteable (Pins) and (iii) mushroom body defect (Mud) [117]. Containing three binding domains for Gαi (GL1, 2 and 3), a Pins molecule becomes activated only when GL3 is occupied by Gαi. Activated Pins in turn recruits Mud to guide spindle alignment in preparation for cell division. It was recently demonstrated that the non-functional GL1 and GL2 domains actually serve as decoy binding sites to sequester Gαi away from GL3, leading to ultrasensitive activation of Pins by Gαi [117]. In the anti-hypoxic stress pathway, factor inhibiting HIF (FIH), which hydroxylates HIF-1α at an asparagine residue, also has a broad range of ankyrin-repeat domain (ARD)-containing proteins as substrate [118]. The competition for FIH between HIF-1α and ARD-containing proteins was predicted to generate switch-like activation of HIF-1α under hypoxia [95].

A variant of substrate competition is the branching point in a metabolic pathway in which two different enzymes compete for the same substrate with vastly different affinities (Michaelis–Menten constants) and metabolize the substrate into two different products. When the fraction of the metabolic flux through the high-affinity enzyme branch is high, the flux through the low-affinity enzyme branch can be ultrasensitive with respect to the substrate supply rate near the point where the high-affinity enzyme is saturated [119]. This is similar to the idea that the stoichiometric inhibitor needs to exist in high abundance for molecular titration to display ultrasensitivity. Recently, this idea of ultrasensitivity arising from flux competition has been extended to translational networks where mRNA molecules belonging to different genes compete for access to a limited pool of ribosomes [120]. Metabolic flux through a futile (substrate) cycle is another example of ultrasensitivity that may be partially explained in the spirit of substrate competition under certain conditions. In this motif, the net flux flowing out of the cycle is sensitive to changes in the forward flux of the cycle when (i) the backward flux of the cycle (which takes mass away from the net flux) is at a level close to the forward flux and (ii) the enzyme catalysing the backward reaction is saturated [121,122].

The quantitative signalling properties of small non-coding RNAs, which repress gene expression by promoting degradation or inhibiting translation of mRNAs, have recently been intensively investigated. Mathematical and experimental studies indicate that small RNAs may regulate gene expression in an ultrasensitive manner by titrating target mRNAs [123–126]. In mammalian cells, the amount of protein translated by the target mRNA exhibited threshold-like response in the presence of a specific microRNA, consistent with a model of molecular titration [127]. Ultrasensitivity through inhibitory titration is also possible with expressed pseudogenes, which may encode for (i) inhibitory proteins that bind to and neutralize functional proteins, or (ii) antisense RNAs that bind to mRNAs and inhibit translation [128].

### 4.5. Covalent modification cycle (zero-order ultrasensitivity)

One of the most prevalent means by which protein activity is regulated is post-translational covalent modification, such as phosphorylation, acetylation and methylation. These covalent modifications affect the affinity of the protein substrate for interacting with other proteins, DNAs or small molecules, and can thus effectively switch the activity of the protein substrate on or off [129]. The modification is usually reversible, involving two opposing processes catalysed by specific enzyme pairs, such as kinase/phosphatase, acetyltransferase/deacetylase and methyltransferase/demethylase. By varying the active/inactive ratio of the protein substrate, the modifier enzyme can regulate its overall activity (figure 3*k*).

Theoretical studies by Goldbeter & Koshland [21,130] three decades ago predicted that when the kinase and phosphatase operate under conditions near saturation by their protein substrates, an ultrasensitive response in substrate phosphorylation can be expected. Known as ‘zero-order ultrasensitivity’ in covalent modification cycle, this prediction was later validated experimentally with a number of signalling proteins and enzymes. The initial evidence came from isocitrate dehydrogenase, an enzyme involved in the Krebs cycle and inhibited by phosphorylation. In an *in vitro* assay system purified from *E. coli*, phosphorylation of isocitrate dehydrogenase exhibited a sigmoid steady-state response that could be partially attributed to zero-order ultrasensitivity [131]. Glycogen phosphorylase, the glycogenolytic enzyme converting glycogen into glucose-1-phosphate, is itself activated by phosphorylation and inactivated by dephosphorylation. Studies of an *in vitro* system containing phosphorylase, phosphorylase kinases and protein phosphatase-1 purified from rabbit skeletal muscles showed that the steady-state level of phosphorylated phosphorylase increased ultrasensitively with a fitted Hill coefficient of 2.35 as the kinase/phosphatase ratio was varied [132,133]. Mathematical modelling strongly suggested that the ultrasensitivity experimentally observed with the activation of AMPK by AMP in INS-1 cells could stem from both multistep signalling as described above and from a zero-order effect owing to possible saturation of AMPKK by AMPK [90]. Similarly, some degree of zero-order ultrasensitivity through covalent modification cycle also plays a role in the activation of MAPK by upstream kinases [24,71]. In addition, MAPK appears to phosphorylate some of its protein substrates with zero-order ultrasensitivity. For instance, Yan, a transcriptional repressor involved in patterning the *Drosophila* embryo, is regulated by MAPK. Phosphorylation of Yan by MAPK promotes its degradation, resulting in a sharp, step-like expression pattern of Yan along the medial–lateral axis in the ventral ectoderm [134]. Zero-order ultrasensitivity does not occur only with protein substrates; conversion of small-molecule substrates, such as NAD and NADH by the enzyme pair formate dehydrogenase and lactate dehydrogenase, can also be switch-like [135].

Zero-order ultrasensitivity associated with covalent modification cycles can be intuitively understood as follows. Consider a system that is at the mid-point steady state with 50 per cent of the protein substrate phosphorylated and the other 50 per cent dephosphorylated. The total amount of the protein is large, far exceeding the Michaelis–Menten constants of the kinase and phosphatase. Both enzymes are thus near being saturated by their respective substrates at the initial steady state. Owing to saturation, both the phosphorylation and dephosphorylation reaction rates are insensitive to changes in substrate concentrations (i.e. zero-order). Because the system is at steady state, the rates of the two opposing reactions are in balance. Now, if the kinase concentration increases slightly, the phosphorylation rate would instantaneously exceed the dephosphorylation rate, causing more protein molecules to become phosphorylated. As the phosphatase is already saturated, any additional increase in the concentration of its substrate (i.e. the phosphorylated protein) would not increase the dephosphorylation rate to counteract the increased phosphorylation rate. Similarly, because the kinase is also saturated, any fractional decrease in the concentration of its substrate (i.e. the dephosphorylated protein) has little effect on the phosphorylation rate. As a result, the net phosphorylation flux would continue until the dephosphorylated protein markedly decreases to a level where the kinase is less saturated, and the phosphorylation rate and the dephosphorylation rate become equal again. A similar but opposite response can be expected when the kinase concentration decreases from the initial mid-point steady state. In either case, a large swing in the concentrations of the phosphorylated and dephosphorylated protein will result, producing a steep response. In the electronic supplementary material, by overlaying the phosphorylation and dephosphorylation rate curves, the mechanism of zero-order ultrasensitivity is illustrated graphically (motif 5).

### 4.6. Positive feedback

A signalling protein can activate itself through positive feedback, which can be in the form of gene auto-regulation, auto-catalysis or through a feedback loop involving intermediate signalling molecules (figure 3*l–m*). A positive feedback loop can behave as a bistable switch if one arm of the loop contains an ultrasensitive motif locally (discussed in §5.1). However, when there is no ultrasensitivity embedded within any arm, the entire loop may function as a monostable URM in response to a stimulatory signal external to the loop. In this case, each arm of the feedback loop may only transfer signal linearly, but ultrasensitivity arises because the signal through the feedback can further activate the molecular species that is directly stimulated by the external signal, thus reinforcing the initial activation. Ultrasensitivity is expected when the external signal and feedback signal impinge on separate but synergistic processes that regulate the common target molecules. In the electronic supplementary material, a mathematical model of auto-catalysis is provided to illustrate how ultrasensitivity can arise with positive feedback (motif 6).

As a common positive feedback motif, gene auto-regulation allows a TF to induce its own transcription. This motif can be frequently found in gene regulatory networks involved in binary lineage specification during development, where attractor states representing different cell types need to be established [136–141]. In theory, auto-regulatory motifs that are not themselves bistable could provide necessary ultrasensitivity for a system containing coherently coupled feedback loops to produce robust bistability [142–145]. Positive feedback regulation in nucleosome modification provides another mechanism for switch-like gene induction by transcriptional activators [146]. In this framework of gene regulation, a TF, after binding to a gene promoter, recruits histone-modifying enzymes. The enzymes modify the chromosomal structure of the local nucleosome to a configuration favouring transcription. The structurally altered nucleosome is also able to recruit additional histone-modifying enzymes to modify nearby nucleosomes to a similar transcription-favouring state. This positive feedback loop, operating locally between histone-modifying enzymes and nucleosomes, has the potential to produce a highly ultrasensitive response in gene activation [146], as well as bistability that allows epigenetic memory [147].

### 4.7. Summary

Each of the six URMs described here has its own unique biochemical, and therefore kinetic, basis for ultrasensitivity. To some extent, the underlying mathematics for positive cooperative binding, multimerization and multistep signalling is similar. The input signal of these three motifs would appear somehow as a power function in the mathematical terms describing the activation process, with the exponent by and large reflecting the number of available binding sites, order of homo-multimers or number of synergistic signalling steps. Molecular titration and zero-order ultrasensitivity take advantage of the dramatic kinetic changes in the molecular binding process near saturation to achieve abrupt responses. For positive feedback loops, the self-reinforcing nature of signalling amplifies the initial activation many more times to produce ultrasensitivity. Biological examples of these ultrasensitive motifs are summarized in table 1.

**Table 1.** Ultrasensitive regulations in molecular signalling networks. *n*_H_, Hill coefficient; n.a., not available.

ultrasensitive regulation	*n*_H_	motif type	reference
signal transduction			
activation of PKA by cAMP	1.4–1.62	(+) cooperative binding	[29,30]
Ca^2+^ binding to calmodulin	1.22–1.33	(+) cooperative binding	[33,34]
Ca^2+^ binding to cPLA2	1.7	(+) cooperative binding	[32]
Ca^2+^ binding to calretinin	3.7	(+) cooperative binding	[31]
activation of PDGFR by PDGF	1.55	homo-dimerization	[46]
activation of Mek-1 by Mos	1.7	multistep signalling zero-order ultrasensitivity	[71]
activation of p42 by Mos	4.9	multistep signalling zero-order ultrasensitivity	[71]
dissociation of Fus3 from ste5 stimulated by α-factor	6	multisite phosphorylation zero-order ultrasensitivity	[148]
activation of CaMKI by Ca^2+^/calmodulin	n.a.	multistep signalling	[86]
activation of CaMKII by Ca^2+^	4.4–8.9	(+) cooperative binding multistep signalling zero-order ultrasensitivity auto-phosphorylation	[149]
activation of Pins by Gαi	3.1	molecular titration	[117]
regulation of transcription factors			
activation of ER by oestradiol	1.1–1.58	homo-dimerization	[52]
activation of PR by progesterone	1.11–1.49	homo-dimerization	[51]
dephosphorylation of NFAT1 by calcineurin	n.a.	multistep signalling (multisite dephosphorylation)	[78]
activation of HIF-1 by low O_2_	n.a.	multistep signalling molecular titration	[95,150]
activation of Nrf2 by ROS	n.a.	multistep signalling gene auto-regulation	[60]
phosphorylation and degradation of Yan by Erk	n.a.	zero-order ultrasensitivity	[134]
transcriptional and translational regulation			
bicoid promoter binding and induction of Hunchback	5	(+) cooperative binding	[42,43]
HSF promoter binding and induction of heat shock proteins	n.a.	(+) cooperative binding	[38,45]
gene induction by CEBPα in the presence of stoichiometric protein inhibitor	1–11.8	molecular titration	[111]
gene induction by tet activators in the presence of decoy DNA binding sites	n.a.	molecular titration	[114]
binding of TATA-binding protein to target sequence in the presence of depleting hairpin DNAs	4.3	molecular titration	[115]
nucleosome modification and recruitment of histone-modifying enzymes	n.a.	positive feedback	[146]
translation of target mRNA in the presence of inhibitory microRNA	n.a.	molecular titration	[127]
regulation of metabolic enzymes and flux			
adenylylation of glutamine synthetase activated by glutamine	5.23	homo-trimerization multistep signalling	[66]
activation of AMPK by AMP	2.5	multistep signalling zero-order ultrasensitivity	[90]
dephosphorylation of isocitrate dehydrogenase by 3-phosphoglycerate	2	multistep signalling zero-order ultrasensitivity	[131]
phosphorylation of phosphorylase	2.35	zero-order ultrasensitivity	[132,133]
conversion between NAD and NADH by FDH and LDH	n.a.	zero-order ultrasensitivity	[135]
metabolism of isocitrate by lyase in the presence of dehydrogenase	n.a.	molecular titration	[119]
cell cycle control			
degradation of Sic1 owing to phosphorylation by Cln-Cdc28	n.a.	multistep signalling (multisite phosphorylation)	[73]
phosphorylation of Cdc25c by Cdk1	2.3	multistep signalling (multisite phosphorylation)	[80]
phosphorylation of Wee1 by Cdk1	3.5	molecular titration (multisite phosphorylation)	[116]

The term ‘cooperativity’ is commonly used in biochemistry to describe synergistic binding events involving multiple molecular subunits, but it is also loosely used in the literature to refer to biochemical processes that generate sigmoid responses through other ultrasensitive mechanisms. Notably, none of the six motifs discussed here seems to produce responses that can be fitted exactly with the Hill function. Moreover, the estimated Hill coefficients of the sigmoid responses are not necessarily true indicators of the maximal local response coefficients. In some cases, the maximal response coefficient can be quite high even though the Hill coefficient is only slightly greater than one (motif 6 in the electronic supplementary material).

Although an individual URM may output steep sigmoid responses, the degree of ultrasensitivity is limited by its kinetic mechanism and the cellular condition under which the motif operates. In the case of positive cooperative binding, multimerization and multistep signalling, the maximal response coefficient depends, respectively, on the number of available binding sites, order of homo-multimers and number of synergistic signalling steps. A steeply sigmoid response through cooperative binding requires multiple binding sites and highly allosteric interactions among these sites, which is structurally challenging. For optimal ultrasensitivity through homo-multimerization where the protein level is transcriptionally regulated, it is preferable for the multimer to be more stable than the monomer [151]. Molecular titration and zero-order ultrasensitivity are in theory capable of producing nearly switch-like responses under appropriate conditions (motifs 4 and 5 in the electronic supplementary material). However, the specific state of the cell *in vivo* may limit their capability. For covalent modification cycles, suboptimal conditions, such as random fluctuation owing to a limited amount of converting enzymes [152], substantial sequestration of protein substrates by the converting enzymes or downstream target proteins [153–155], spatial separation of the opposing converting enzyme pairs [156], and existence of converting enzymes that are not strongly irreversible [157], may compromise the degree of zero-order ultrasensitivity.

Consequently, it is common for multiple URMs to be arranged together in signalling networks to achieve robustly steep responses. The effect is similar to volume amplification by connecting preamplifiers and power amplifiers in an audio system. The maximal response coefficient of the combined motifs can ideally approach the product of the maximal response coefficients of the individual motifs. Motif combination may also provide functional robustness [158], as the loss or weakening of ultrasensitivity in one motif owing to gene mutation may only partially compromise the overall ultrasensitivity [159]. A canonical example of motif combination is the MAPK signalling cascade, which exhibits increasing response sigmoidicity moving down the cascade [71,160]. The MAPK cascade ultrasensitivity can be attributed primarily to three mechanisms: (i) multistep signalling through dual phosphorylation; (ii) zero-order ultrasensitivity; and (iii) three-tiered structure of the cascade to multiply the ultrasensitivity achieved at each tier. Recently, it was also suggested that kinase cascading itself might be another possible source of increased sigmoidicity when enzyme distribution among intermediate complexes is explicitly considered [161]. Combination of various URMs is also found in many other signalling processes, such as induction of antioxidants by oxidative stressors [60], and Ca^2+^ activation of Ca^2+^/calmodulin-dependent protein kinase II (CaMKII) that underlies long-term potentiation and memory formation in the hippocampus [149].

## 5. Ultrasensitivity and complex network dynamics

Complex dynamics of molecular signalling networks arise collectively from interactions among individual components. Multistability, adaptation, oscillation and chaos are common examples of network dynamics. Higher-level cellular functions, such as proliferation, differentiation, homeostasis, mobility, metabolism and rhythmic behaviours, require proper integration of these dynamical properties across a multitude of intricate biochemical networks. Using bistability, adaptation and oscillation, we illustrate below the importance of signal amplification conferred by ultrasensitivity in rendering these dynamics from properly structured networks.

### 5.1. Bistability

Many cellular-level responses, including proliferation, differentiation, lineage specification and apoptosis, are all-or-none, in which cells choose between two discrete outcomes. Once cells commit to one fate over the other, the state transition is usually irreversible under physiological conditions. Gene and protein networks capable of bistability underpin the discreteness and irreversibility of many of these all-or-none responses [13,162]. Bistability generally requires two conditions: (i) the network topology must be positive and/or double-negative feedback loops; and (ii) at least one arm of the feedback loop must embed motifs that can transfer signal ultrasensitively [13,162,163].

**Figure 4.**
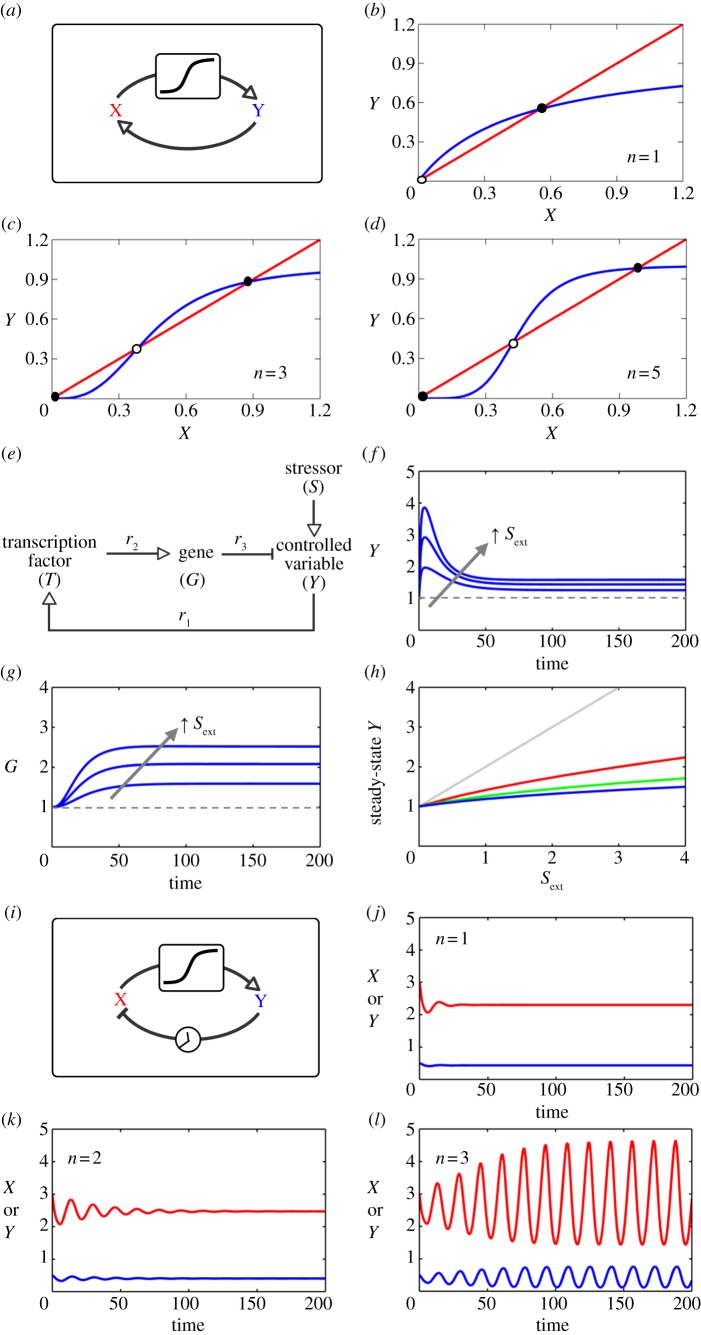
(*Overleaf*.) Illustration of the roles of ultrasensitivity for complex network dynamics. (*a*–*d*) Ultrasensitivity is required for bistability. (*a*) Gene *X* and *Y* form a double-positive feedback loop, where *X* activates *Y* in an ultrasensitive manner, and *Y* activates *X* in a Michaelis–Menten manner. The system is described by equations (5.1) and (5.2), and the parameters are *k*_1_ = 3, *k*_2_ = 1, *k*_3_ = 1, *k*_4_ = 1, *K_x_* = 2, *K_y_* = 0.5 and *n* = 1, 3 or 5. (*b*–*d*) Stability analysis using nullclines with different *n-*values. The intersection points between *X* (red) and *Y* (blue) nullclines indicate the steady states of the feedback system (solid dot, stable steady state; empty dot, unstable steady state). The system is bistable when there are three intersection points: two stable steady states and one unstable steady state in between (*c*) and (*d*). The *Y* nullclines in (*c*) and (*d*) show increasing degree of ultrasensitivity, making bistability arise easily. Reducing ultrasensitivity makes the *X* and *Y* nullclines difficult to intersect three times, leading to monostability, as illustrated in (*b*). (*e*–*h*) Ultrasensitivity helps negative feedback loops to achieve robust cellular adaptation and homeostasis. (*e*) A generic negative feedback circuit underlying cellular adaptation and homeostasis against stress. *S* represents the total stress level containing background/internal stress (*S*_bkg_) and external stress (*S*_ext_), thus *S* = *S*_bkg_ + *S*_ext_. The system is described by equations (5.5)–(5.7), and the default parameters are *k*_1_ = 1, *k*_2_ = 1, *k*_3_ = 0.1, *k*_4_ = 0.1, *k*_5_ = 1.01, *k*_6_ = 0.01, *S*_bkg_ = 1 and *n* = 2. (*f*,*g*) Adaptive response of controlled variable *Y* and underlying induction of anti-stress gene *G* under persistent external stress at various levels (*S*_ext_ = 1, 2 and 3). Dashed lines are baseline levels of *Y* and *G* in the absence of *S*_ext_. (*h*) Adapted steady-state levels of *Y* with respect to various levels of *S*_ext_. In the open-loop case (*R*_loop_ = 0), the response is linear (grey line). As *R*_loop_ increases by setting Hill coefficient *n* = 1, 2 and 3, the respective response (red, green and blue curves) becomes increasingly subsensitive, indicating improved adaptation and more robust homeostasis. To maintain the same basal level of *G*, *k*_5_ = 0.02, 0.11, 1.01 and 10.01 for *n* = 0, 1, 2 and 3, respectively. (*i*–*l*) Ultrasensitivity is required for a negative feedback loop to generate sustained oscillation. (*i*) Genes *X* (red) and *Y* (blue) form a negative feedback loop, where *X* activates *Y* in an ultrasensitive manner, and *Y* inhibits *X* linearly with a time delay. The system is described by equations (5.10) and (5.11), and the parameters are *k*_1_ = 1, *k*_2_ = 1, *k*_3_ = 1, *k*_4_ = 1, *K* = 3, *τ* = 5 and *n* = 1, 2 or 3. *τ* denotes the time delay from *Y* to *X*. Initial *X* = 3 and *Y* = 0.5. (*j*–*l*) As the Hill coefficient *n* increases from 1 to 3, the feedback system tends to oscillate better. Small *n-*values only give rise to damped oscillation, whereas large *n-*values lead to sustained oscillation.

The ultrasensitivity requirement can be illustrated graphically with a simple two-variable system in which genes *X* and *Y* activate each other transcriptionally, with linear degradation of each gene product (figure 4*a*). *Y* activates *X* in a simple Michaelis–Menten fashion, whereas *X* activates *Y* ultrasensitively, as described by the Hill function:5.1
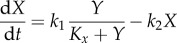

and5.2
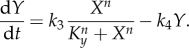
The possible steady states of this system appear as intersection points of the *X* and *Y* nullclines, which are obtained by setting d*X*/d*t* and d*Y*/d*t* to zero (figure 4*b*–*d*):5.3
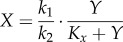


and5.4
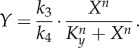


For the system to be bistable, the two nullclines must intersect each other three times, corresponding to two stable steady states and one unstable steady state in between [163]. Given that the *X* nullcline bends upward or is at best a straight line, the *Y* nullcline has to be sufficiently ‘twisted’ in a certain way in order to cross the *X* nullcline back and forth multiple times. This behaviour can be readily achieved when the *Y* nullcline is sigmoid (i.e. when *Y* responds to *X* in a typical ultrasensitive manner; figure 4*c*–*d*). The higher the degree of sigmoidicity of the *Y* nullcline (achieved by increasing the Hill coefficient *n* here), the more robust the bistability. Conversely, if the *Y* nullcline is not ultrasensitive, the system only has a single stable steady state (figure 4*b*). On the other hand, if the *X* nullcline is sigmoid, the ultrasensitivity requirement for the *Y* nullcline could be relaxed, still permitting three intersection points. Thus, a certain degree of ultrasensitivity in either of the two arms of a positive feedback loop is essential for bistability to arise. In addition to the graphical argument, the requirement of ultrasensitivity for bistability can be captured more formally by examining the eigenvalues of the Jacobian matrix of the feedback system, or, for a metabolic pathway, by examining the ratio of the feedback elasticity and the degradation elasticity of the product exerting the feedback [164,165].

### 5.2. Adaptation and cellular homeostasis

To survive, biological organisms must be able to adapt to a fluctuating environment and maintain a relatively stable internal milieu in both tissues and cells. At the cellular level, many physical and chemical variables (such as cell size, ions and oxygen) are maintained at a relatively constant set-point. Likewise, potentially deleterious intracellular molecules such as reactive oxygen species (ROS), mis-folded proteins and toxic metals have to be kept within certain healthy ranges.

Although a feed-forward mechanism can be useful, negative feedback regulation is primarily responsible for robust cellular adaptation and homeostasis [14,166]. Figure 4*e* illustrates a general homeostatic control scheme against cellular stress. Cellular stressor *S* (*S* = background/internal stressor *S*_bkg_ + external stressor *S*_ext_) increases the level of controlled variable *Y*. Changes in the level of *Y* are sensed either directly or indirectly by transcription factor *T*, which in turn induces anti-stress gene *G* that functions to counteract changes in *Y*. In a simple form such a feedback system can be described by the following ordinary differential equations:5.5
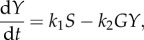
5.6
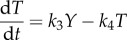
5.7
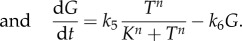


Such a model system can simulate a typical adaptive response. At the onset of the stress, *Y* first spikes up and then it gradually returns to a steady-state level close to the baseline in the continuous presence of the stress (figure 4*f*). Adaptation occurs because the anti-stress gene *G* is slowly upregulated by *T* during the process (figure 4*g*). In the absence of feedback control, *Y* is assumed to increase linearly with *S*. To understand how ultrasensitivity modulates the steady-state *Y* versus *S* response, we need to calculate the systems-level response coefficient 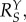 which is5.8
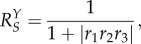
according to Kholodenko *et al.* [6] and Zhang & Andersen [14]. Here, *r*_1_, *r*_2_ and *r*_3_ are local response coefficients (gain) for the regulation of *T* by *Y*, *G* by *T* and *Y* by *G*, respectively. Because the feedback loop gain *R*_loop_ = |*r*_1_*r*_2_*r*_3_| ≥ 0, and thus 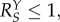 the steady-state *Y* versus *S* response is mostly subsensitive, appearing concave downward (figure 4*h*). Strong homeostatic performance requires a small 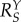, which in turn requires a high loop gain. In the specific example here, a high loop gain can be achieved by increasing Hill coefficient *n* in the term describing the ultrasensitive induction of *G* by *T*. This way, the percentage increase in the adapted steady-state level of *Y* becomes much smaller than the increase in stressor *S*. Multiple URMs with large *r*_1_, *r*_2_ and *r*_3_ can accomplish a high loop gain, thus ultrasensitivity helps negative feedback loops to achieve robust adaptation and homeostasis against external perturbations. Similarly, feedback control through allosteric inhibition of enzymes by downstream products can also be found in metabolic pathways to maintain flux or metabolite homeostasis [167,168].

For the negative feedback circuit in figure 4*e*, it is worth noting that the steady-state expression level of anti-stress gene *G* with respect to stressor *S* is governed by the following systems-level response coefficient:5.9
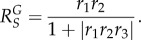


If the loop gain 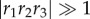 and is primarily due to large *r*_1_ and/or *r*_2_, while *r*_3_ ≈ −1, then 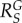 tends to approach unity, suggesting a linear relationship between *G* and *S*. Thus a negative feedback circuit embedding highly ultrasensitive motifs may improve, counter-intuitively, the linearity of cell signalling. Indeed, in the signal transduction pathway involving the ultrasensitive MAPK cascade, which is encompassed in various negative feedback loops [169], phosphorylation of ERK (in NIH 3T3 cells) exhibited a nearly linear relationship with the extracellular stimuli [170], a result that was predicted by an earlier computational study [16]. Similarly, engineered negative gene auto-regulation circuits harbouring high-degree cooperativity within the feedback loop in yeast cells have been demonstrated to output linearized expression of reporter genes with respect to the inducer concentration [171].

### 5.3. Oscillation

Many biological rhythms originate at the cellular level, with oscillating periods ranging widely from seconds to days. Examples include spontaneous action potential in cardiac pacemaker cells, pulsatile hormone secretion from endocrine cells and the circadian clock in the suprachiasmatic nucleus neurons. Cells also exhibit oscillatory dynamics in response to external perturbations, such as cytosolic Ca^2+^ spikes stimulated by activation of G-protein-coupled receptors, sustained p53 pulses triggered by double strand DNA breaks, damped NF-κB oscillation stimulated by lipopolysaccharide and damped oscillatory response to iron stress in *E. coli* [172–175]. While oscillation may arise from positive feedback with substrate depletion [176], most cellular oscillatory behaviours require negative feedback as the primary network structure. For sustained oscillation, the negative feedback topology has to be complemented with two additional conditions: (i) time delay in signalling and (ii) ultrasensitivity [177,178].

A simple two-variable negative feedback system with time delay (figure 4*i*), described by the following two ordinary differential equations, is used here to illustrate the role of ultrasensitivity:5.10
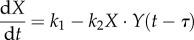


and5.11
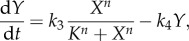
where *X* activates *Y* ultrasensitively as represented by the Hill function, and *Y* inhibits *X* by promoting its degradation in a linear fashion but with a time delay *τ*. The role of ultrasensitivity and time delay for oscillation can be intuitively understood as follows. A key kinetic property of biochemical processes, which invariably involve molecular binding and unbinding, is that steady states are usually approached asymptotically in time. Therefore, for a linear negative feedback system (where *X* activates *Y* linearly and *Y* inhibits *X* linearly), as *X* rises and falls, *Y* would never be able to rise and fall by exactly the same amplitude as *X* (same fold change, to be exact, from peak to trough), even given sufficient time. This would result in a pulse of *Y* of smaller amplitude than the preceding *X* pulse. By the same token, the smaller *Y* pulse would in turn lead to an even smaller *X* pulse, and so on. Thus, a linear negative feedback system can at best exhibit damped oscillation. In a nonlinear feedback system, where *X* can activate *Y* ultrasensitively, a pulse of *X* can result in a pulse of *Y* of larger amplitude owing to signal amplification. This larger *Y* pulse is then likely to promote a subsequent *X* pulse of equal or even higher amplitude than the previous *X* pulse even if *Y* only regulates *X* linearly. The non-diminishing *X* pulse allows the process to repeat itself, resulting in sustained oscillation. Thus, ultrasensitivity compensates for the inherent loss of pulse amplitude occurring in a linear system (figure 4*j*–*l*). Increasing the time delay by increasing the number of intermediate steps in the feedback loop generally relaxes the requirement for the degree of ultrasensitivity and vice versa [20,179,180]. It was long predicted that the intrinsically ultrasensitive MAPK cascade, when operating in a negative feedback loop, may bring about sustained oscillations [181]. More recently, Shankaran *et al*. [182] indeed observed that phosphorylation of ERK in the nucleus and cytoplasm of human mammary epithelial cells stimulated by EGF is robustly oscillatory, with pulse frequencies comparable with those predicted by the earlier MAPK oscillation model. Finally, ultrasensitivity is also required in the so-called relaxation oscillator, which contains essentially a negative feedback loop and a nested positive feedback loop. In this circuit, the positive feedback loop with embedded ultrasensitivity provides a reversible bistable switch, whereas the negative feedback loop functions to drive the switch on and off periodically [178].

## 6. Concluding remarks

In the new millennium, as the connection details of large molecular signalling networks are increasingly mapped out, understanding their dynamical behaviours has become the new challenge in biological research. Similar to the way engineers learn how electrical circuits function, biologists need to first discover and understand small network motifs and recurring sub-networks before undertaking the task of making sense of more complex biological networks. URMs, characterized by a sigmoid I/O relationship, can arise through a variety of kinetic mechanisms. As central to cellular processes as transistors are to modern electronics, URMs are the basic biochemical signal amplifiers necessary for complex molecular networks to generate bistability, adaptation/homeostasis, oscillation and other nonlinear dynamics. The discovery and characterization of these network motifs will continue to help bring systems-level perspectives to the quantitative investigation of existing and newly discovered biochemical pathways and their attendant cellular outcomes.

## 7. Acknowledgements

We would like to thank the financial support from NIEHS-P42ES04911, NIEHS-R01ES016005, NIEHS-R01ES020750, Dow Chemical Company, ExxonMobil Foundation and the Long-Range Research Initiative of the American Chemistry Council for supporting this work. The authors have declared that there are no conflicts of interest.

## Supplementary Material

Mathematical Models of Ultrasensitive Motifs

## Supplementary Material

SBML Models
