# Supplementary material for: Ultrasensitive response motifs: basic amplifiers in molecular signalling networks
Source: Open Biol. 2013 Apr;3(4):130031. doi: 10.1098/rsob.130031 (PMC3718334; doi:10.1098/rsob.130031)
Supplement: Mathematical Models of Ultrasensitive Motifs [file rsob130031-s1.pdf]

Supplementary Materials: Mathematical Models of Ultrasensitive Motifs

Ultrasensitive Response Motifs: Basic Amplifiers in Molecular Signaling Networks

Qiang Zhang, Sudin Bhattacharya, and Melvin E. Andersen

Motif 1: Positive Cooperative Binding

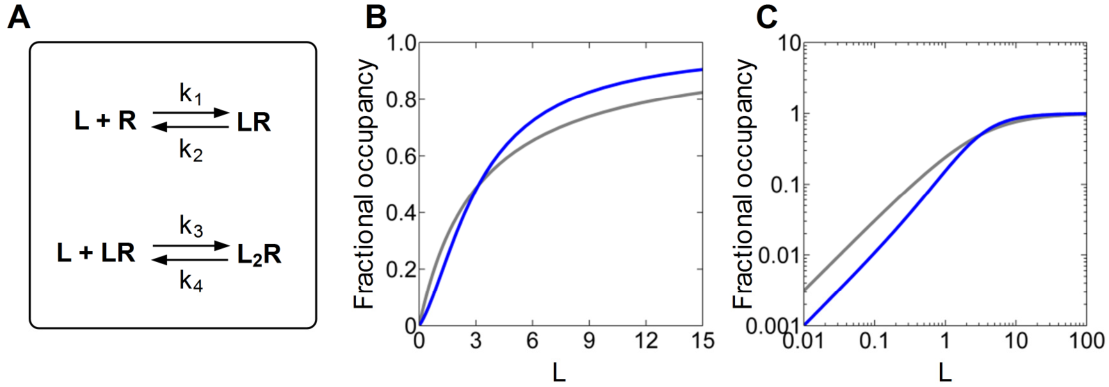

In this positive cooperative binding model (panel A), signaling molecule L binds to a target receptor molecule R which has two identical binding sites for L. LR represents R with one binding site occupied by L and L_2R represents R with both binding sites occupied by L. The overall activity of R is proportionately correlated to its fractional occupancy by L. The binding processes can be described mathematically as follows:

Equations:

$$d[LR]/dt = 2\cdot k_1\cdot[L]\cdot[R] - k_2\cdot[LR] - k_3\cdot[L]\cdot[LR] + 2\cdot k_4\cdot[L_2R] \quad (1.1)$$

$$d[L_2R]/dt = k_3\cdot[L]\cdot[LR] - 2\cdot k_4\cdot[L_2R] \quad (1.2)$$

$$[R] = R_{tot} - [LR] - [L_2R] \quad (1.3)$$

$$\text{Fractional occupancy} = ([LR] + 2\cdot[L_2R])/(2\cdot R_{tot}) \quad (1.4)$$

Parameters: k_1=0.01; k_2=0.1; k_3=0.01; k_4=0.01; R_{tot}=100 (total amount of receptor molecules).

Positive cooperative binding occurs because the binding affinity of the second binding (k_3/k_4=1) is 10-fold higher than the first binding (k_1/k_2=0.1). The resulting steady-state fractional occupancy of R vs. L is a slightly sigmoid curve with Hill coefficient=1.48 (panel B: blue curve). Panel C shows the stimulus-response curve on a log-log scale. The Michaelis-Menten function serves as a reference response (gray curve).

Motif 2: Homo-dimerization

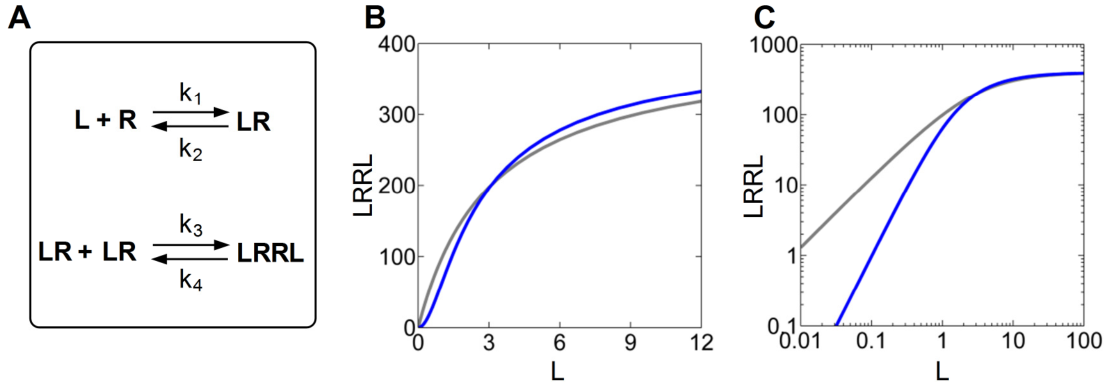

In this homo-dimerization model (panel A), ligand L binds to receptor R forming LR. Two LRs then come together to form a homo-dimer LRRL. The binding processes can be described mathematically as follows:

Equations:

$$d[LR]/dt = k_1 \cdot [L] \cdot [R] - k_2 \cdot [LR] - 2 \cdot k_3 \cdot [LR] \cdot [LR] + 2 \cdot k_4 \cdot [LRRL] \quad (2.1)$$

$$d[LRRL]/dt = k_3 \cdot [LR] \cdot [LR] - k_4 \cdot [LRRL] \quad (2.2)$$

$$[R] = R_{tot} - [LR] - 2 \cdot [LRRL] \quad (2.3)$$

Parameters: k_1=0.01; k_2=0.1; k_3=0.001; k_4=0.1; R_{tot}=1000 (total amount of receptor molecules).

According to the law of mass action, the association rate of two LRs to form an LRRL is proportional to the square of the concentration of LR. This step is the place where ultrasensitivity arises in this motif. The resulting steady-state LRRL vs. L response is a slightly sigmoid curve with Hill coefficient= 1.31 (panel B: blue curve). When plotted on a log-log scale, the response coefficient (measured by the slope) is as high as 2 for low concentrations of L (panel C). The Michaelis-Menten function serves as a reference response (gray curve).

Motif 3: Multistep Signaling

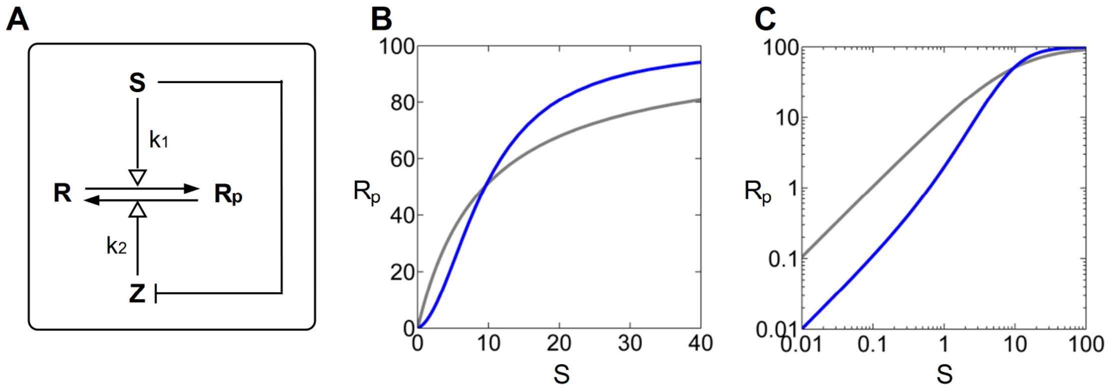

In this multistep signaling example (panel A), kinase S regulates target protein R in two ways. S (i) directly activates R by phosphorylating R to R_p, and (ii) indirectly inhibits dephosphorylation of R_p to R by inhibiting the phosphatase Z. The phosphorylation and counterbalancing dephosphorylation processes can be described mathematically as follows:

Equations:

$$d[R_p]/dt = k_1 \cdot [S] \cdot [R] - k_2 \cdot [Z] \cdot [R_p] \qquad (3.1)$$

$$[R] = R_{tot} - [R_p] \qquad (3.2)$$

$$[Z] = Z_{max}/(1+[S]/K) \qquad (3.3)$$

Parameters: k_1=0.01; k_2=0.01; K=1; R_{tot}=100 (total amount of dephosphorylated R and phosphorylated R_p); Z_{max}=100 (maximum amount of active phosphatase Z).

By simultaneously activating phosphorylation directly and inhibiting dephosphorylation indirectly through inhibiting Z, S regulates R_p through coherently controlling the two reversible modification steps. The resulting steady-state R_p vs. S response is a sigmoid curve with Hill coefficient=1.88 (panel B: blue curve). Panel C shows the stimulus-response curve on a log-log scale. The Michaelis-Menten function serves as a reference response (gray curve).

Motif 4: Molecular Titration

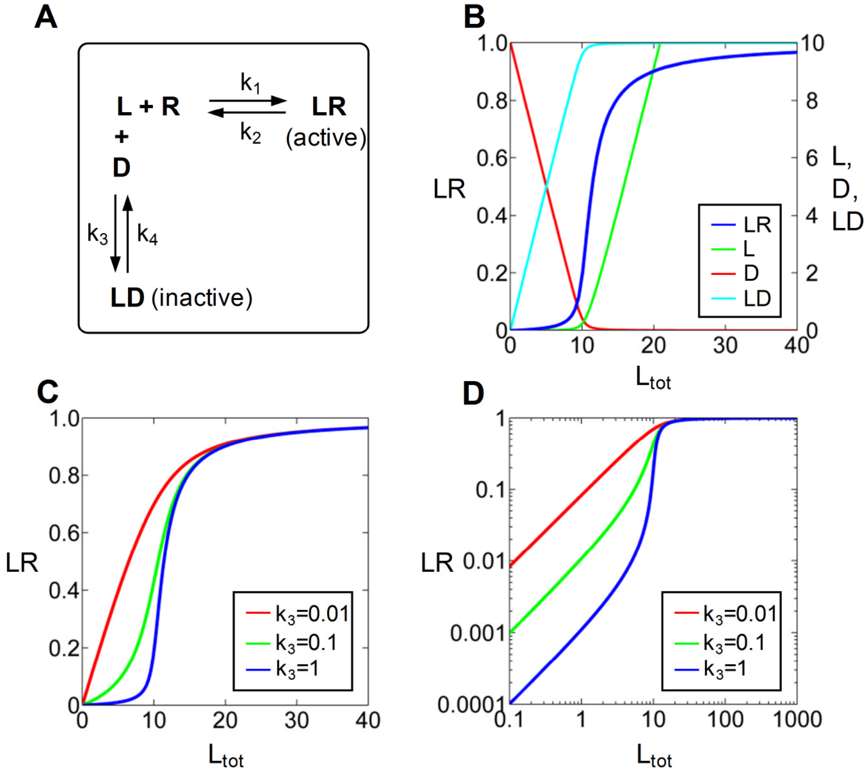

In this molecular titration example (panel A), ligand L reversibly binds either to cognate receptor R forming an active complex LR or to decoy receptor D forming an inactive complex LD. Thus D competes with R for L by titrating L away from R. The stimulus-response evaluated here is the steady-state level of LR vs. total L (free L and those in LR and LD complex). The competitive binding processes can be described mathematically as follows:

Equations:

$$d[LR]/dt = k_1\cdot[L]\cdot[R] - k_2\cdot[LR] \quad (4.1)$$

$$d[LD]/dt = k_3\cdot[L]\cdot[D] + k_4\cdot[LD] \quad (4.2)$$

$$[L] = L_{tot} - [LR] - [LD] \quad (4.3)$$

$$[R] = R_{tot} - [LR] \quad (4.4)$$

$$[D] = D_{tot} - [LD] \quad (4.5)$$

Parameters: k_1=0.01; k_2=0.01; k_3=1; k_4=0.01; R_{tot}=1 (total amount of receptor including R and LR); D_{tot}=10 (total amount of decoy receptor including D and LD).

The decoy receptor D exists in large excess compared with cognate receptor R and has a higher affinity for ligand L than R does. Thus when $L_{tot}<D_{tot}$, most of L molecules in the system will be sequestered by D to form inactive complex LD (panel B: cyan curve), with few free L molecules (panel B: green curve) left available for binding to R. As more L is added to the system and L_{tot} approaches a level comparable to D_{tot}, most D molecules would be used up by L by forming LD, with few free D molecules left (panel B: red curve). At that point any additional L molecules further added into the system will be almost all available for binding to R rather than being titrated away by D. This kinetic shift at the point where D is saturated by L would cause an abrupt increase in the level of free L and consequently LR, resulting in an ultrasensitive response for steady-state LR vs. L_{tot} (panel B: blue curve, Hill coefficient=5.89). The higher the binding affinity between L and the decoy receptor D, the higher the degree of ultrasensitivity. This effect is illustrated by varying k_3, the association rate constant between L and D in the two bottom panels (panel C: linear scale, panel D: log-log scale).

Motif 5: Zero-order Covalent Modification Cycle

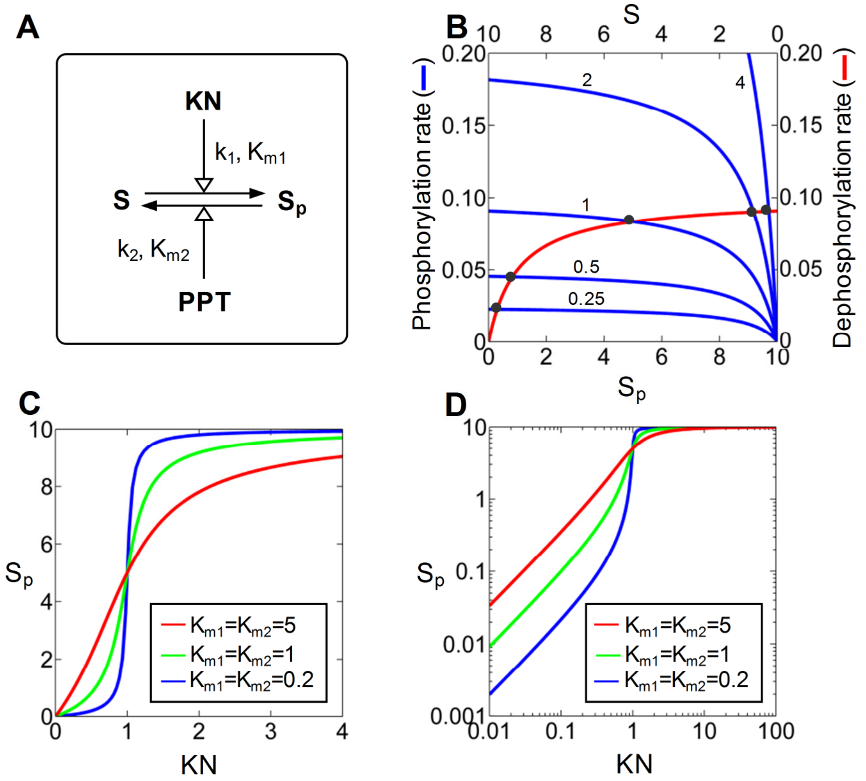

Covalent modification cycle through protein phosphorylation and dephosphorylation is used in this model to illustrate the effect of zero-order ultrasensitivity (panel A). The processes of protein substrate S being phosphorylated by kinase KN into S_p and S_p being dephosphorylated by phosphatase PPT are described mathematically as follows:

Equations: $d[S_p]/dt = k_1 \cdot [KN] \cdot [S]/(K_{m1}+[S]) - k_2 \cdot [PPT] \cdot [S_p]/(K_{m2}+[S_p])$ (5.1)

$[S] = S_{tot} - [S_p]$ (5.2)

Parameters: $k_1=0.1$; $k_2=0.1$; $K_{m1}=1$; $K_{m2}=1$; $[PPT]=1$; $S_{tot}=10$ (total amount of S and S_p).

The concentration of PPT is constant, and that of KN is varied to change the phosphorylation rate. Panel B shows the basis of ultrasensitivity in this motif, where the dephosphorylation rate (red curve) and phosphorylation rate (blue curve) as functions of S_p (or S) concentration are plotted. Each blue curve is obtained at a particular KN concentration (which is indicated by the number next to the blue curve). Intersection points (solid black dots) between blue and red curves indicate equal phosphorylation and dephosphorylation rates, i.e., the steady state. Because $S_{tot}>>K_{m1}=K_{m2}$, KN and PPT work at nearly saturated conditions for most concentrations of S and S_p, respectively, and the blue and red curves thus appear quite flat for the most part. As a result, when [KN] = [PPT] = 1, the blue and red curves intersect very tightly, i.e., near their saturation zones. Because of this tight intersection, even a small increase or decrease in KN concentration, which either pushes up or down the blue curve, would cause a large swing of the intersection point horizontally. This behavior results in an abrupt change in the steady-state S_p or S concentrations with respect to KN and thus an ultrasensitive response (panel C: green curve, Hill coefficient=3.8). The degree of saturation of the two converting enzymes KN and PPT by their substrates dictates the degree of ultrasensitivity. At $K_{m1}=K_{m2}=0.2$, where KN and PPT are further saturated, the response becomes much more ultrasensitive (panel C: blue curve, Hill coefficient=13.4). At $K_{m1}=K_{m2}=5$, KN and PPT are much less saturated, the response becomes much less ultrasensitive (panel C: red curve, Hill coefficient=1.68). Panel D shows the stimulus-response curves on a log-log scale.

Motif 6: Positive Feedback

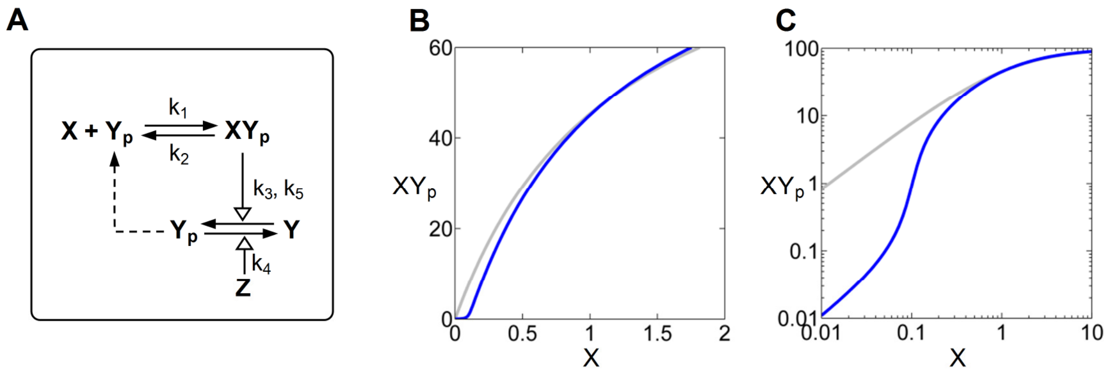

In this positive feedback example (panel A), a signaling protein X partners with a phosphorylated protein Y_p to form an active kinase complex XY_p. XY_p can phosphorylate other free Y molecules, thus completing a positive auto-catalysis loop. Z is a phosphatase that dephosphorylates Y_p. These processes can be mathematically described as follows:

Equations:

$d[XY_p]/dt = k_1 \cdot [X] \cdot [Y_p] - k_2 \cdot [XY_p]$ (6.1)

$d[Y_p]/dt = - k_1 \cdot [X] \cdot [Y_p] + k_2 \cdot [XY_p] + k_3 \cdot [XY_p] \cdot [Y] + k_5 \cdot [Y] - k_4 \cdot [Z] \cdot [Y_p]$ (6.2)

$[Y] = Y_{tot} - Y_p - XY_p$ (6.3)

Parameters: k_1=0.1; k_2=0.1; k_3=0.1; k_4=0.1; k_5=0.01 (basal rate constant of phosophorylation independent of XY_p); Y_{tot}=100 (total amount of Y, Y_p, and XY_p); [Z]=10.

Ultrasensitivity occurs because as X increases, the amount of Y_p which partners with X also increases due to increased formation of XY_p which autophosphorylates Y. This self-reinforcing cycle continues until a new steady state is reached. The steady-state XY_p vs. X response appears as a threshold response (panel B: blue curve, Hill coefficient=1.14). However, on a log-log scale, the maximal local response coefficient, as measured by the slope, is close to 5 (panel C: blue curve), indicating strong ultrasensitivity. The Michaelis-Menten function serves as a reference response (gray curve).
